# Vibration Analysis of Multilayer Stepped Cross-Sectional Carbon Nanotubes

**DOI:** 10.3390/nano15201550

**Published:** 2025-10-11

**Authors:** Yunus Onur Yildiz, Murat Sen, Osman Yigid, Mesut Huseyinoglu, Sertac Emre Kara

**Affiliations:** 1 Department of Naval Architecture and Marine Engineering, Maritime Faculty, Bursa Technical University, 16310 Bursa, Türkiye; onur.yildiz@btu.edu.tr; 2 Department of Mechanical Engineering, Faculty of Engineering, Firat University, 23119 Elazig, Türkiye; 3 Department of Mechanical Engineering, Faculty of Engineering and Architecture, Bitlis Eren University; 13100 Bitlis, Türkiye; oyigid@beu.edu.tr; 4 Department of Mechanical Engineering, Faculty of Engineering, Dicle University, 21280 Diyarbakir, Türkiye

**Keywords:** modal analysis, molecular dynamics, natural frequency, carbon nanotube, multilayer carbon nanotubes

## Abstract

This study comprehensively investigates the dynamic vibration behavior of multilayer carbon nanotubes with stepped cross-sectional geometries under various boundary conditions, which is crucial for their advanced engineering applications. The methodology integrates classical molecular dynamics simulations to determine the bending stiffness of single-walled and multi-walled atomistic structures, which are subsequently utilized in the Euler–Bernoulli beam theory based on nonlocal elasticity for vibration analysis. The research focuses on elucidating the influence of the *μ*/*L* ratio (a key length parameter) and different support conditions on the natural frequencies and mode shapes of these nanostructures. Key findings reveal that the cross-sectional geometry significantly impacts the vibrational characteristics. A consistent trend observed across all examined boundary conditions is a decrease in natural frequencies as the *μ*/*L* ratio increases, indicating that increased free length or reduced fixed length leads to lower stiffness and, consequently, reduced natural frequencies. The study presents Frequency Response Functions (FRFs) and the first four mode shapes, which visually confirm these dynamic characteristics. Graphical representations further reinforce the sensitivity of natural frequencies to both the *μ*/*L* ratio and support conditions. The systematic analysis presented in this work provides vital data for predicting resonance phenomena, optimizing structural stability, and enabling precise control over the vibrational response of these advanced nanomaterials in diverse engineering applications.

## 1. Introduction

Carbon nanotubes (CNTs), celebrated for their exceptional mechanical, electrical, and thermal properties, have become building blocks in nanotechnology, finding applications ranging from nanocomposites to nanoelectronics. These fascinating structures, characterized by a hexagonal arrangement of carbon atoms, exhibit remarkable properties stemming from their unique structural configuration. The unique structure of carbon nanotubes, encapsulating a one-dimensional volume of space, contributes to their remarkable electronic and mechanical characteristics [1,2,3]. Carbon nanotube-based structures are emerging as promising candidates for various applications, including sensors, actuators, and energy storage devices [4].

The mechanical properties of CNTs are significantly influenced by their diameter, chirality, and number of walls [5]. The aspect ratio and high elastic modulus of carbon nanotubes also facilitate the design of composite materials with large strain differences between constituents, further enhancing energy dissipation ability [6].

Molecular dynamics simulations have emerged as a powerful computational tool for investigating the dynamic behavior of materials at the atomic and molecular level, offering valuable insights into their properties and performance. Molecular dynamics simulations are used to simulate the interactions between atoms and molecules by integrating Newton’s equations of motion. These simulations provide a detailed understanding of the dynamic processes occurring within the system, allowing researchers to investigate phenomena that are difficult or impossible to observe experimentally. Molecular dynamics simulations have been used to investigate the mechanical properties of carbon nanotubes, providing insights into their stiffness, strength, and deformation mechanisms [7]. These simulations have also been used to study the vibrational behavior of carbon nanotubes, revealing information about their vibrational modes and frequencies. By simulating the dynamic behavior of carbon nanotubes under different conditions, researchers can gain a deeper understanding of their properties and how they can be tailored for specific applications. The accurate modeling of carbon nanotubes requires considering the interatomic interactions and the complex geometry of the structure. Molecular dynamics simulations provide atomic-level details of the vibrational behavior of carbon nanotubes. Elasticity in both single- and multi-walled nanotubes (MWCNTs) is determined by their elastic modulus, a property that can be experimentally assessed using techniques such as transmission electron microscopy [8]. Utilizing 3D multiscale finite element models of representative volume elements enables probabilistic analysis of carbon nanotube-reinforced polymer composite materials, offering a framework for determining dynamic reliability and hazard based on modal parameters [9]. Indeed, a comprehensive understanding of the mechanical behavior of nanomaterials is paramount for their successful engineering implementation. For carbon nanotubes, capturing size-dependent flexural behaviors often necessitates the use of advanced simulation and continuum models beyond classical theories [10,11,12], such as nanoscale continuum mechanics and micropolar elasticity models. These sophisticated approaches, coupled with molecular dynamics simulations, are crucial for accurately validating material parameters and overcoming the limitations of traditional characterization techniques. Analyzing the vibration modes of carbon nanotubes is crucial for understanding their dynamic behavior and ensuring their reliability in diverse applications. Modal vibration tests, which involve measuring modal frequencies, damping factors, and mode shapes, offer a rapid and cost-effective approach to characterizing the elastic and viscoelastic properties of these materials [13]. The cross-sectional geometry of carbon nanotube beams significantly influences their vibrational characteristics, affecting both the natural frequencies and mode shapes. Variations in diameter and chirality alter the stiffness and mass distribution of the nanotube, leading to changes in the resonant frequencies [14]. Şimşek [15] presented an analytical method for the forced vibrations of a system of two identical, elastically connected carbon nanotubes carrying a moving nanoparticle, based on the nonlocal elasticity theory. The validity of the presented method was verified by comparing the results with those obtained using the Galerkin method and the Newmark time integration method. Belhadj et al. [16] used the differential quadrature method (DQM), a semi-analytical method, to investigate the bending vibrations of a single-walled carbon nanotube (SWCNT). Hussain et al. [17] conducted a dynamic analysis of SWCNTs based on the Donnell thin shell theory. They used a wave propagation approach to derive the characteristic frequency equation that defines the natural vibration frequencies, and they compared their results with those obtained from molecular dynamics simulations. Liu and Wang [18] investigated the nonlinear thermal vibration behavior of an SWCNT using molecular dynamics simulation and a nonlinear, nonplanar beam model. They observed that the large-amplitude vibrations of SWCNTs, resulting from increasing temperature, lead to geometric nonlinearity and the presence of whirling motion accompanied by energy transfer between flexural motions. Hossain and Lellep [19] investigated the vibration characteristics of a cracked, conical, and elastically connected identical twin nanobeam. They used the Euler–Bernoulli beam theory and Eringen’s nonlocal elasticity theory. They explored how the crack depth, crack location, nonlocal parameters, conicity ratio, and spring constant affect the dynamic behavior of the system. Liu and Wang [20] investigated the significance of quantum effects in the thermal vibration of SWCNTs. They reported that the simulation results from Semi-Quantum Molecular Dynamics (SQMD) only showed agreement with the nonlocal Timoshenko beam model incorporating quantum effects (TBQN). Consequently, they demonstrated that quantum and nonlocal effects must be necessarily incorporated into the modeling for an accurate analysis of SWCNT vibrations, particularly under conditions of low temperature, short length, and high-order modes. In ref. [21], molecular dynamics simulations and continuum analyses based on the Euler–Bernoulli and Timoshenko beam theories were conducted for double-walled carbon nanotubes (DWNTs), considering the effects of nanotube length and chirality. It was stated that the surrounding matrix stiffness and the radial displacement between the nanotubes significantly affect the resonance frequencies. Horng [22] investigated the feasibility of using SWCNTs as mass sensors. To this end, he presented an analytical solution for the analysis of the resonant frequencies and mode shapes of a cantilever beam with a rigid particle attached at its free end. He examined how the mass and volume of the added particle cause shifts in the resonant frequencies and explored how this relationship could be utilized to determine the mass and volume of the added particle.

The potential of carbon nanotubes to enhance the mechanical properties of composite materials has spurred research into their use in structural applications. The functionalization of carbon nanotubes can improve their dispersion and interfacial adhesion with the matrix material, further enhancing the mechanical properties of the composite. There is a need for more studies to elucidate the underlying mechanisms governing the vibration behavior of carbon nanotube beams with different cross-sectional geometries and under various environmental conditions. With this motivation, the ultimate goal of this study is to investigate the vibration behavior of these materials. For this purpose, firstly, atomistic models of SWCNT and MWCNT structures are generated. Then, the numerical specimens are subjected to four-point bending by employing classical molecular dynamics simulations. The obtained bending stiffnesses from molecular dynamics simulations are used to analyze the vibration characteristics of carbon nanotubes with variable cross-sections through Euler–Bernoulli beam theory based on nonlocal elasticity.

## 2. Methodology

The methodology relies on molecular dynamics (MD) analysis to calculate the bending stiffness of carbon nanotubes (SWCNTs and MWCNTs), and the Euler–Bernoulli beam theory based on nonlocal elasticity for vibration analysis.

### 2.1. Molecular Dynamics Analysis

The molecular dynamics analyses provide the bending stiffness of both single- and multi-walled carbon nanotubes. For this purpose, the atomistic models have to be generated using VMD (Visual Molecular Dynamics) [23]. As is known, carbon nanotubes are modeled as a single layer of a graphene sheet rolled into a seamless hollow cylinder, and they are categorized as armchair (*m* = *n*), zigzag (*n* = 0), or chiral (*m* ≠ *n*). These chiral indices (*n*, *m*) specify the nanotube’s structure and geometry. On the other hand, MWCNTs were generated as concentric SWCNTs, with the number of walls ranging from 2 to 5. The interlayer spacing in MWCNTs is generally considered similar to that of graphene, approximately 0.34 nm [24]. MWCNT structures are characterized by a sequence of SWCNTs with increasing chiral indices from the innermost to the outermost layer, ensuring a consistent interlayer distance. In this way, the atomistic models are generated with the geometric properties given in Table 1.

**Figure 1:**
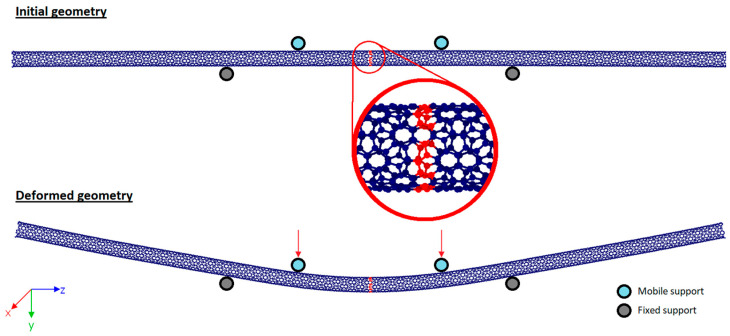
The four-point bending test scheme.

In order to comprehensively evaluate the bending stiffness of CNTs, the four-point bending test procedure was implemented using molecular dynamics simulations. Although the four-point bending test is commonly used at the macroscale, nanoscale studies can also be found in the literature [10,26]. The test employed two mobile rigid supports as loading points and two fixed rigid supports, as shown in Figure 1. This method is an accurate way to imitate pure bending deformation for specimens [27]. The distance between fixed support points is 20 nm, while the distance between mobile support points is 10 nm. These distance values were chosen arbitrarily because they did not affect the desired energy curvature curve [10]. Furthermore, the movement of the atoms shown in red in Figure 1 is restricted in the *x*- and *z*-directions in order to prevent the test specimen subjected to bending from sliding in these directions.

**Figure 2:**
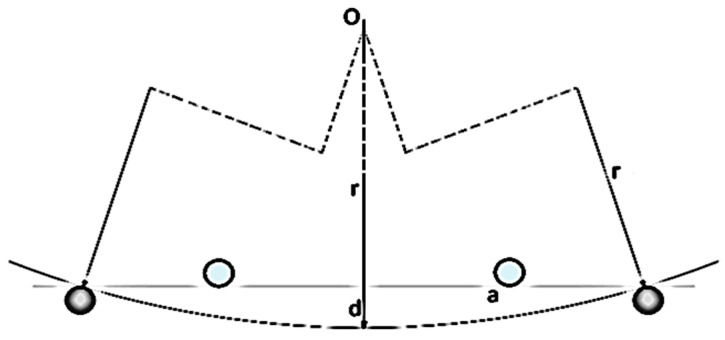
Schematic of the curvature determination.

One of the main parameters for the 4-point bending test is the curvature value, which can be determined from Pythagoras’ theorem as given in Figure 2. The curvature is determined for each time step using Equation (1) as follows:

(1) $$\kappa =\frac{1}{r}=\frac{2d}{d^{2}+a^{2}}$$

where *κ* is the curvature, *r* is the radius, *a* is the distance between the mid-point of the bending specimen and the fixed support point, and *d* is the distance from the central axis. The value *d* is determined based on the mean of the y-coordinates of the atoms encircled in red in Figure 1.

The elastic bending energy of the test specimen is determined by Equation (2) as follows:

(2) $$∆U=\int_{0}^{L}\frac{1}{2}D\kappa^{2}ds$$

where ∆*U* is the bending energy, *D* is the bending stiffness (*D* = *EI*, where *E* is Young’s modulus and *I* is the moment of inertia), *s* is local coordinates along the curve, and *L* is the length of the beam. The equation can be assumed to have constant curvature and bending stiffness for our case.

(3) $${∆U}_{L}=\frac{∆U}{L}=\frac{1}{2}D\kappa^{2}$$

All molecular dynamics simulations were performed using the Large-scale Atomic/Molecular Massively Parallel Simulator (LAMMPS) open-source software [28]. The adaptive intermolecular reactive empirical bond order (AIREBO) potential with a cutoff distance of 3Å was utilized to describe carbon–carbon atomic interactions [29,30,31]. This potential is well-established for its accuracy in capturing bond interactions, breaking, and formation in carbon nanomaterials. Initial atomic models are subjected to minimization in order to mitigate potential internal stresses. Furthermore, minimized structures were relaxed under an isothermal–isobaric (NPT) ensemble using a Nosé–Hoover thermostat at zero pressure to reach a state of thermal equilibrium. All these simulations were generally carried out at 10 K to suppress the impact of thermal fluctuations. A consistent time step of 1 femtosecond (fs) was typically used for time integration.

### 2.2. Numerical Analysis

**Figure 3:**
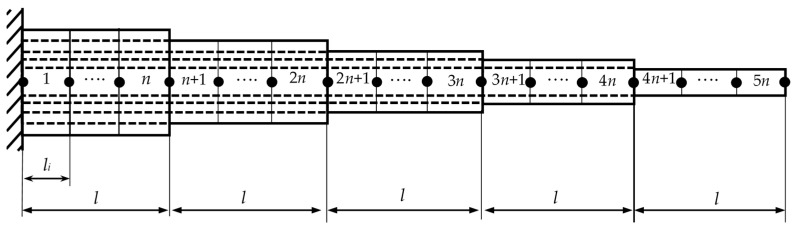
The variable cross-section carbon nanotube model with clamped–free boundary conditions.

A numerical solution was performed within the framework of the Euler–Bernoulli beam theory based on nonlocal elasticity to obtain the vibration characteristics of carbon nanotubes with stepped cross-sections. A visual representation of the carbon nanotube with a stepped cross-section is given in Figure 3.

The model presented in Figure 3 consists of 5 steps, each with an equal length of *l* and a total beam length of *L*. From the fixed end to the free end, the number of walls of the carbon nanotube decreases, becoming single-walled at the outermost free step. Each layer is further divided into *n* elements, with each element having a length of *l_i_*. The resulting model, therefore, consists of a total of 5*n* finite elements and 5*n* + 1 nodes.

The fundamental equation of motion for a transversely vibrating Euler–Bernoulli beam can be expressed as follows:

(4) $$\frac{\partial^{2}}{\partial^{2}x}\left(EI(x)\frac{\partial^{2}w(x,t)}{\partial x^{2}}\right)+\rho A(x)\frac{\partial^{2}w(x,t)}{\partial t^{2}}=0$$

**Figure 4:**
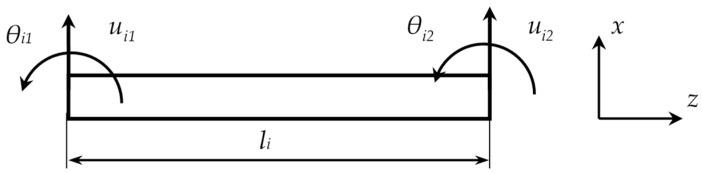
Euler–Bernoulli beam element and its DoFs.

Here, *E*, *I*, *ρ*, and *A* represent the modulus of elasticity, area moment of inertia, density, and cross-sectional area of the beam, respectively. According to the Euler–Bernoulli beam theory, degrees of freedom (DoFs) for each element are shown in Figure 4.

In the beam element shown in Figure 4, $u_{i1}$ and $u_{i2}$ represent the translational DoFs, while $\theta_{i1}$ and $\theta_{i2}$ denote the rotational DoFs of the ith element. Therefore, the coordinates for each element can be expressed as follows:

(5) $$\left\{q_{i}\right\}={\left\{u_{i1},\theta_{i1},u_{i2},\theta_{i2}\right\}}^{T}$$

Each node has 2 degrees of freedom, and each beam element has a total of 4 degrees of freedom. The element-based stiffness and mass matrices obtained for each finite element (FE) can be expressed as follows.

The element-based stiffness matrix of the nanotube (*i* = 1, 2, … , 5*n*) is

(6) $${\left[k\right]}^{\left(i\right)}=\left[\begin{array}{cccc} k_{11}^{\left(i\right)} & \begin{array}{l} k_{12}^{\left(i\right)} \end{array} & k_{13}^{\left(i\right)} & k_{14}^{\left(i\right)}\\ k_{21}^{\left(i\right)} & k_{22}^{\left(i\right)} & k_{23}^{\left(i\right)} & k_{24}^{\left(i\right)}\\ k_{31}^{\left(i\right)} & k_{32}^{\left(i\right)} & k_{33}^{\left(i\right)} & k_{34}^{\left(i\right)}\\ k_{41}^{\left(i\right)} & k_{42}^{\left(i\right)} & k_{43}^{\left(i\right)} & k_{44}^{\left(i\right)} \end{array}\right]=\frac{E_{i}I_{i}}{l_{i}^{3}}\left[\begin{array}{cccc} 12 & 6l_{i} & -12 & 6l_{i}\\ 6l & {4l_{i}}^{2} & -6l_{i} & {2l_{i}}^{2}\\ -12 & -6l_{i} & 12 & -6l_{i}\\ 6l_{i} & {2l_{i}}^{2} & -6l_{i} & {4l_{i}}^{2} \end{array}\right]$$

Here, *E_i_*, *I_i_*, and *l_i_* represent the modulus of elasticity, area moment of inertia, and length of each beam element, respectively. The term ${\left[k\right]}^{\left(i\right)}$ denotes the stiffness matrix of the corresponding element.

The element-based mass matrix of the nanotube (i = 1, 2, … , 5*n*) is

(7) $${\left[m\right]}^{\left(i\right)}=\left[\begin{array}{cccc} m_{11}^{\left(i\right)} & \begin{array}{l} m_{12}^{\left(i\right)} \end{array} & m_{13}^{\left(i\right)} & m_{14}^{\left(i\right)}\\ m_{21}^{\left(i\right)} & m_{22}^{\left(i\right)} & m_{23}^{\left(i\right)} & m_{24}^{\left(i\right)}\\ m_{31}^{\left(i\right)} & m_{32}^{\left(i\right)} & m_{33}^{\left(i\right)} & m_{34}^{\left(i\right)}\\ m_{41}^{\left(i\right)} & m_{42}^{\left(i\right)} & m_{43}^{\left(i\right)} & m_{44}^{\left(i\right)} \end{array}\right]=\frac{\rho_{i}A_{i}l_{i}}{420}\left[\begin{array}{cccc} 156 & 22l_{i} & 54 & -13l_{i}\\ 22l_{i} & {4l_{i}}^{2} & 13l_{i} & {-3l_{i}}^{2}\\ 54 & 13l_{i} & 156 & -22l_{i}\\ -13l_{i} & {-3l_{i}}^{2} & -22l_{i} & {4l_{i}}^{2} \end{array}\right]$$

In this context, *ρ_i_*, *A_i_*, and ${\left[m\right]}^{\left(i\right)}$ represent the mass density, cross-sectional area, and mass matrix of the associated beam element, respectively.

**Figure 5:**
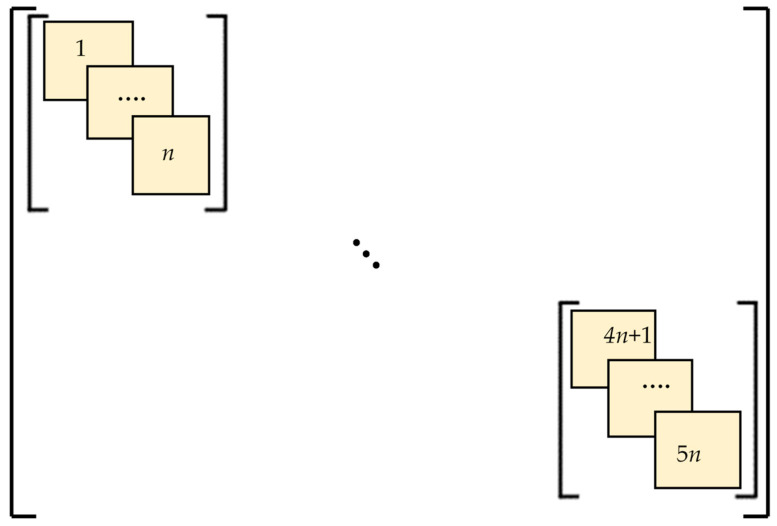
Assembly of the element matrices for a variable cross-section nanotube.

Once all the element matrices of the beam have been defined, the global stiffness and mass matrices of the stepped cross-section nanotube must be obtained by assembling these matrices. The stepped beam values of *EI* and *ρA* change for each step, as indicated in Equation (8). The assembly procedure is illustrated in Figure 5.

(8) $$EI=\left\{\begin{array}{ccc} {\left(EI\right)}_{1} & if & 0\leq x\leq l\\ {\left(EI\right)}_{2} & if & l\leq x\leq 2l\\ {\left(EI\right)}_{3} & if & 2l\leq x\leq 3l\\ {\left(EI\right)}_{4} & if & 3l\leq x\leq 4l\\ {\left(EI\right)}_{5} & if & 4l\leq x\leq L \end{array}\right.,\;\rho A=\left\{\begin{array}{ccc} {\left(\rho A\right)}_{1} & if & 0\leq x\leq l\\ {\left(\rho A\right)}_{2} & if & l\leq x\leq 2l\\ {\left(\rho A\right)}_{3} & if & 2l\leq x\leq 3l\\ {\left(\rho A\right)}_{4} & if & 3l\leq x\leq 4l\\ {\left(\rho A\right)}_{5} & if & 4l\leq x\leq L \end{array}\right.$$

The global stiffness and mass matrices for the 5n-element stepped beam described above are given below. Since the created global matrices have a total of 5*n* + 1 nodes, and each node has two coordinates (one rotational and one translational), each resulting global matrix will have a DoF of 2(5*n* + 1).

The nanotube’s global stiffness and mass matrices can be constructed as follows:

(9) $$\left[K\right]=\left[\begin{array}{ccccccccccc} k_{11}^{\left(1\right)} & k_{12}^{\left(1\right)} & k_{13}^{\left(1\right)} & k_{14}^{\left(1\right)} & 0 & 0 & 0 & 0 & 0 & 0 & 0\\ k_{21}^{\left(1\right)} & k_{22}^{\left(1\right)} & k_{23}^{\left(1\right)} & k_{24}^{\left(1\right)} & 0 & 0 & 0 & 0 & 0 & 0 & 0\\ k_{31}^{\left(1\right)} & k_{32}^{\left(1\right)} & \left(k_{33}^{\left(1\right)}+k_{11}^{\left(2\right)}\right) & \left(k_{34}^{\left(1\right)}+k_{12}^{\left(2\right)}\right) & k_{13}^{\left(2\right)} & k_{14}^{\left(2\right)} & 0 & 0 & 0 & 0 & 0\\ k_{41}^{\left(1\right)} & k_{42}^{\left(1\right)} & \left(k_{43}^{\left(1\right)}+k_{21}^{\left(2\right)}\right) & \left(k_{44}^{\left(1\right)}+k_{22}^{\left(2\right)}\right) & k_{23}^{\left(2\right)} & k_{24}^{\left(2\right)} & 0 & 0 & 0 & 0 & 0\\ 0 & 0 & k_{31}^{\left(2\right)} & k_{32}^{\left(2\right)} & \left(k_{33}^{\left(2\right)}+\ldots \right) & \left(k_{34}^{\left(2\right)}+\ldots \right) & \ldots & \ldots & 0 & 0 & 0\\ 0 & 0 & k_{41}^{\left(2\right)} & k_{42}^{\left(2\right)} & \left(k_{43}^{\left(2\right)}+\ldots \right) & \left(k_{44}^{\left(2\right)}+\ldots \right) & \ldots & \ldots & 0 & 0 & 0\\ 0 & 0 & 0 & 0 & \ldots & \ldots & \ldots & \ldots & 0 & 0 & 0\\ 0 & 0 & 0 & 0 & \ldots & \ldots & \ldots & \left(k_{33}^{\left(5n-1\right)}+k_{11}^{\left(5n\right)}\right) & \left(k_{34}^{\left(5n-1\right)}+k_{12}^{\left(5n\right)}\right) & k_{13}^{\left(5n\right)} & k_{14}^{\left(5n\right)}\\ 0 & 0 & 0 & 0 & 0 & 0 & 0 & \left(k_{43}^{\left(5n-1\right)}+k_{21}^{\left(5n\right)}\right) & \left(k_{44}^{\left(5n-1\right)}+k_{22}^{\left(5n\right)}\right) & k_{23}^{\left(5n\right)} & k_{24}^{\left(5n\right)}\\ 0 & 0 & 0 & 0 & 0 & 0 & 0 & k_{31}^{\left(5n\right)} & k_{32}^{\left(15\right)} & k_{33}^{\left(5n\right)} & k_{34}^{\left(5n\right)}\\ 0 & 0 & 0 & 0 & 0 & 0 & 0 & k_{41}^{\left(5n\right)} & k_{42}^{\left(15\right)} & k_{43}^{\left(5n\right)} & k_{44}^{\left(5n\right)} \end{array}\right]$$

(10) $$\left[M\right]=\left[\begin{array}{ccccccccccc} m_{11}^{\left(1\right)} & m_{12}^{\left(1\right)} & m_{13}^{\left(1\right)} & m_{14}^{\left(1\right)} & 0 & 0 & 0 & 0 & 0 & 0 & 0\\ m_{21}^{\left(1\right)} & m_{22}^{\left(1\right)} & m_{23}^{\left(1\right)} & m_{24}^{\left(1\right)} & 0 & 0 & 0 & 0 & 0 & 0 & 0\\ m_{31}^{\left(1\right)} & m_{32}^{\left(1\right)} & \left(m_{33}^{\left(1\right)}+m_{11}^{\left(2\right)}\right) & \left(m_{34}^{\left(1\right)}+m_{12}^{\left(2\right)}\right) & m_{13}^{\left(2\right)} & m_{14}^{\left(2\right)} & 0 & 0 & 0 & 0 & 0\\ m_{41}^{\left(1\right)} & m_{42}^{\left(1\right)} & \left(m_{43}^{\left(1\right)}+m_{21}^{\left(2\right)}\right) & \left(m_{44}^{\left(1\right)}+m_{22}^{\left(2\right)}\right) & m_{23}^{\left(2\right)} & m_{24}^{\left(2\right)} & 0 & 0 & 0 & 0 & 0\\ 0 & 0 & m_{31}^{\left(2\right)} & m_{32}^{\left(2\right)} & \left(m_{33}^{\left(2\right)}+\ldots \right) & \left(m_{34}^{\left(2\right)}+\ldots \right) & \ldots & \ldots & 0 & 0 & 0\\ 0 & 0 & m_{41}^{\left(2\right)} & m_{42}^{\left(2\right)} & \left(m_{43}^{\left(2\right)}+\ldots \right) & \left(m_{44}^{\left(2\right)}+\ldots \right) & \ldots & \ldots & 0 & 0 & 0\\ 0 & 0 & 0 & 0 & \ldots & \ldots & \ldots & \ldots & 0 & 0 & 0\\ 0 & 0 & 0 & 0 & \ldots & \ldots & \ldots & \left(m_{33}^{\left(5n-1\right)}+m_{11}^{\left(5n\right)}\right) & \left(m_{34}^{\left(5n-1\right)}+m_{12}^{\left(5n\right)}\right) & m_{13}^{\left(5n\right)} & m_{14}^{\left(5n\right)}\\ 0 & 0 & 0 & 0 & 0 & 0 & 0 & \left(m_{43}^{\left(5n-1\right)}+m_{21}^{\left(5n\right)}\right) & \left(m_{44}^{\left(5n-1\right)}+m_{22}^{\left(5n\right)}\right) & m_{23}^{\left(5n\right)} & m_{24}^{\left(5n\right)}\\ 0 & 0 & 0 & 0 & 0 & 0 & 0 & m_{31}^{\left(5n\right)} & m_{32}^{\left(5n\right)} & m_{33}^{\left(5n\right)} & m_{34}^{\left(5n\right)}\\ 0 & 0 & 0 & 0 & 0 & 0 & 0 & m_{41}^{\left(5n\right)} & m_{42}^{\left(5n\right)} & m_{43}^{\left(5n\right)} & m_{44}^{\left(5n\right)} \end{array}\right]$$

After obtaining the mass and stiffness matrices for the variable cross-section beam, the equation of motion for the system’s free vibrations becomes

(11) $$\left[M\right]\left\{\ddot{x}\right\}+\left[K\right]\left\{x\right\}=\left\{0\right\}$$

Assume that the harmonic behavior mode shapes and natural frequencies of the beam can be obtained from the above identity as follows:

(12) $$\left(\left[K\right]-\omega_{i}^{2}\left[M\right]\right)\left\{\phi_{i}\right\}=\left\{0\right\}$$

Here, $\phi_{i}$ is the mode shapes and $\omega_{i}$ is the natural frequency of the ith mode.

### 2.3. The Core of Nonlocal Constitutive Relation

The nonlocal model hinges on Eringen’s constitutive relation [32], which redefines how stress relates to strain. Instead of the simple local relationship (stress at a point depends only on strain at that point), the nonlocal stress, $\sigma_{nonlocal}$, is related to the local stress, $\sigma_{local}$, through a differential equation. For a one-dimensional case like a beam, there is the following:

(13) $$\sigma_{local}=\sigma_{nonlocal}-{\left(e_{0}a\right)}^{2}\frac{\partial^{2}\sigma_{nonlocal}}{\partial x^{2}}$$

where $\sigma_{nonlocal}$ is the nonlocal stress of interest. $\sigma_{local}=E\varepsilon$ is the classical stress from Hooke’s law, where *E* is Young’s modulus and *ε* is the strain. $e_{0}a$ is the nonlocal parameter, representing a small internal characteristic length of the material, a is the internal characteristic length, and $e_{0}$ is a constant. This parameter is the source of all size-dependent effects. Rearranging this relation gives the fundamental equation:

(14) $$\sigma_{non-local}-{\left(e_{0}a\right)}^{2}\frac{\partial^{2}\sigma_{nonlocal}}{\partial x^{2}}=E\varepsilon$$

The axial strain $\varepsilon_{xx}$ at a distance y from the neutral axis is given by the beam’s curvature as follows:

(15) $$\varepsilon_{xx}=-y\frac{\partial^{2}w}{\partial x^{2}}$$

The bending moment *M* is the integral of the stress over the cross-sectional area *A* as follows:

(16) $$M=\int_{A}\sigma_{xx}ydA$$

Applying the nonlocal constitutive equation to the axial stress *σ_xx_* yields

(17) $$\sigma_{xx}-{\left(e_{0}a\right)}^{2}\frac{\partial^{2}\sigma_{xx}}{\partial x^{2}}=E(-y\frac{\partial^{2}w}{\partial x^{2}})$$

To obtain the bending moment, one integrates this entire equation over the cross-section, multiplying the following by *y*:

(18) $$\int_{A}\sigma_{xx}ydA-{\left(e_{0}a\right)}^{2}\int_{A}\frac{\partial^{2}\sigma_{xx}}{\partial x^{2}}ydA=-E\int_{A}y^{2}\frac{\partial^{2}w}{\partial x^{2}}dA$$

The first term is the definition of the nonlocal bending moment, *M*. In the second term, we can move the derivative $\frac{\partial^{2}}{\partial x^{2}}$ outside the integral, making it ${\left(e_{0}a\right)}^{2}\frac{\partial^{2}}{\partial x^{2}}\int_{A}\sigma_{xx}ydA$, which is simply ${\left(e_{0}a\right)}^{2}\frac{\partial^{2}M}{\partial x^{2}}$. In the third term, $\frac{\partial^{2}w}{\partial x^{2}}$ is constant over the area, and $\int_{A}y^{2}dA$ is the definition of the second moment of area, *I*. This term becomes $-EI\frac{\partial^{2}w}{\partial x^{2}}$, which is the definition of the classical (local) bending moment, $M_{local}$. This gives us the crucial nonlocal moment–curvature relationship

(19) $$M-{\left(e_{0}a\right)}^{2}\frac{\partial^{2}M}{\partial x^{2}}=M_{local}=-EI\frac{\partial^{2}w}{\partial x^{2}}$$

The classical equation for the dynamics of a beam (from force and moment balance) relates the bending moment to the transverse acceleration as follows:

(20) $$\frac{\partial^{2}M}{\partial x^{2}}=\rho A\frac{\partial^{2}w}{\partial t^{2}}$$

Rearranging the nonlocal moment equation to solve for *M* results in the following:

(21) $$M=M_{local}+{\left(e_{0}a\right)}^{2}\frac{\partial^{2}M}{\partial x^{2}}$$

Substituting the dynamic relation into this obtains the following:

(22) $$M=M_{local}+{\left(e_{0}a\right)}^{2}\rho A\frac{\partial^{2}w}{\partial t^{2}}$$

Finally, we take the second partial derivative with respect to x of this entire equation as follows:

(23) $$\frac{\partial^{2}M}{\partial x^{2}}=\frac{\partial^{2}M_{local}}{\partial x^{2}}+{\left(e_{0}a\right)}^{2}\frac{\partial^{4}M}{\partial x^{2}\partial t^{2}}$$

Substitute the dynamic relation on the left and the definition of *M_local_* on the right as follows:

(24) $$\rho A\frac{\partial^{2}w}{\partial t^{2}}=\frac{\partial^{2}}{\partial x^{2}}\left(-EI\frac{\partial^{2}w}{\partial x^{2}}\right)+{\left(e_{0}a\right)}^{2}\rho A\frac{\partial^{4}w}{\partial x^{2}\partial t^{2}}$$

Rearranging gives the final governing equation for free vibration of a nonlocal beam as follows:

(25) $$EI\frac{\partial^{4}w}{\partial x^{4}}+\rho A\left(\frac{\partial^{2}w}{\partial t^{2}}-{\left(e_{0}a\right)}^{2}\frac{\partial^{4}w}{\partial x^{2}\partial t^{2}}\right)=0$$

For free vibration, $\left(w\left(x,t\right)=W\left(x\right)e^{i\omega t}\right)$, the equation of motion of the transverse displacement becomes

(26) $$EI\frac{\partial^{4}W}{\partial x^{4}}-\omega^{2}\rho A\left(W-{\left(e_{0}a\right)}^{2}\frac{\partial^{2}W}{\partial x^{2}}\right)=0$$

Multiplying Equation (26) by a test function *δw*(*x*), integrating the domain [0, *L*], and then applying integration by parts yields the weak form as follows:

(27) $$\int_{0}^{L}{EIW}^{\prime \prime }(x,t){\delta W}^{\prime \prime }(x)dx-\omega^{2}\int_{0}^{L}\rho AW(x,t)\delta W(x)dx-\omega^{2}\mu^{2}\int_{0}^{L}{\rho AW}^{\prime }(x,t){\delta W}^{\prime }(x)dx+Boundary\;Terms=0$$

Here, $\mu =e_{0}$a, and in the above relation, all conventional boundary terms vanish [33].

*N* is the shape functions of the nanotube, and from this weak form, one can identify three matrices in the FEM discretization.

(28) $$K_{local}=\int_{0}^{L}EI{\left(B_{2}\right)}^{T}B_{2}dx$$

where $B_{2}=d^{2}N/dx^{2}$ relates nodal DOFs to curvature. *K_local_* is the classical bending stiffness matrix mentioned above (Equation (9)).

(29) $$M_{local}=\int_{0}^{L}\rho AN^{T}Ndx$$

(30) $$G=\int_{0}^{L}\rho A{\left(B_{1}\right)}^{T}B_{1}dx$$

Here, $B_{1}=dN/dx$ relates nodal DOFs to slope. *M_local_* is the classical mass matrix mentioned above (Equation (10)), and *G* is called the slope-based (gradient inertia) matrix. As the cross-section of the stepped nanotube changes, a numerical 3-point Gaussian integration is applied to the *G* element matrix [34].

After assembling all elements, the semi-discrete equation of motion becomes

(31) $$K_{i}\phi_{i}=\omega_{i}^{2}(M_{i}+\mu^{2}G_{i})\phi_{i}$$

Equation (31) indicates that setting the parameter *μ* to zero reduces the model to the local Euler–Bernoulli beam theory. The natural frequencies of a stepped beam, whose elements may have varying properties, are found by solving the generalized eigenvalue problem formed after assembling the global *K*, *M*, and *G* matrices and applying the boundary conditions. The following three boundary conditions are investigated in the current work:

$$\mathrm{clamped}\mathrm{end}\Rightarrow w=0,\;\;\frac{dw}{dx}=0$$

simplysupportedend⇒w=0,  M=0

freeend⇒M=0,  V=0

## 3. Results and Discussion

**Figure 6:**
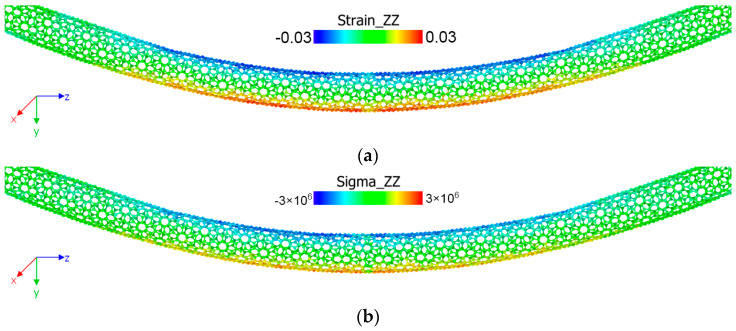
Schematic of the curvature determination: (**a**) strain distribution; (**b**) stress distribution.

MD simulations were performed to obtain the bending stiffness (*EI*) values for each stepped nanotube. These values were then utilized in numerical analysis simulations to determine the dynamic properties of the nanotube. In the MD simulation, the main aim is to obtain the bending stiffness from the molecular dynamics analyses. For this purpose, the four-point bending tests are carried out for SWCNTs and MWCNTs. As a result of these molecular dynamics analyses, some mechanical parameters, such as strain and stress tensors, can be obtained inherently. Figure 6a illustrates the distribution of the atomic Green–Lagrangian strain tensor (*ε_zz_*) [35,36,37] of the SWCNT at the “*d*” displacement (“*d*” is referred to in Figure 2) of 2 nm. According to Figure 6, compression occurs in the upper part of the SWCNT, while tension appears in the lower part. It is a typical pure bending behavior for beam structures. However, as the test progresses, surface buckling is observed in the sample, which is attributed to the structure of carbon nanotubes. For the same geometry and time span, the atomic stress tensor (*σ_zz_*) is also illustrated in Figure 6b. The stress tensor is calculated using the virial stress theorem by Zhou [38].

**Figure 7:**
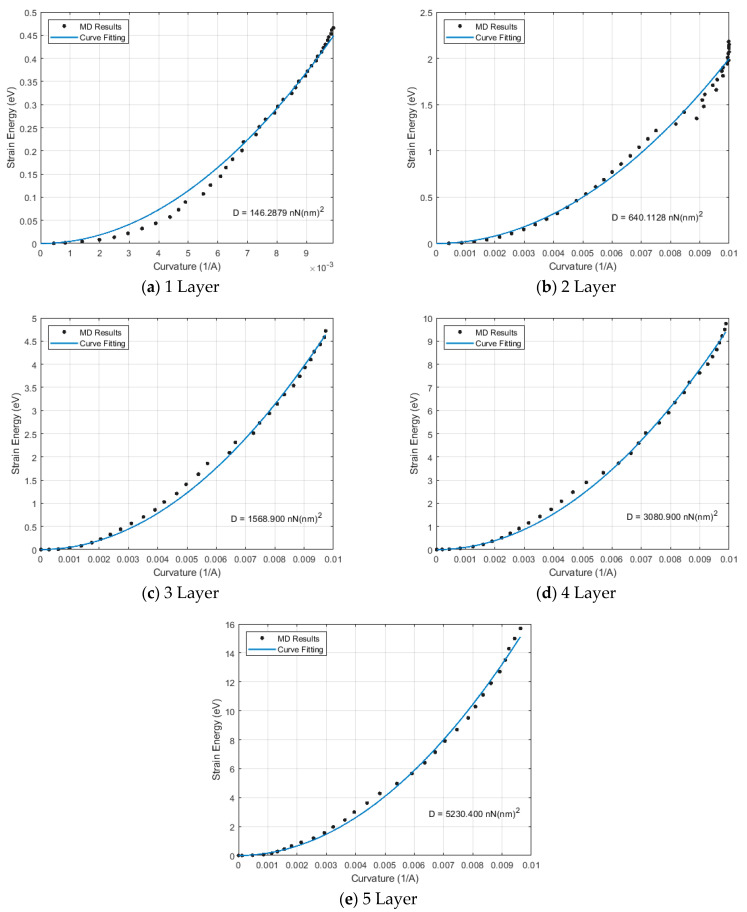
Bending properties of all atomistic models.

A parabolic correlation exists between strain energy and curvature, as formulated in Equation (3). Using this relationship, we can fit a curve to match the strain energy and curvature results obtained from molecular dynamics analyses. Using this defined curve, the primary objective, “the bending stiffness” for each atomistic model, can be obtained as shown in Figure 7.

The mechanical properties used in the creation of mass and stiffness matrices in calculations using Euler–Bernoulli beam theory are given in Table 2.

The nanotube model was created by dividing each segment into 20 elements, with each segment having *l* = 50 nm. Natural frequencies and mode shapes were calculated by solving the eigenvalue problem using the general mass and general stiffness matrices created by considering nonlocal elasticity. Table 3 presents the natural frequencies of the first four vibration modes for *μ*/*L* = 0 with clamped–free boundary conditions. As shown in Table 3, the natural frequencies of the first four modes of the undamped nanotube range from 0.2 to 3.5 GHz.

Frequency Response Functions (FRFs) can be calculated using eigenvalues (squared natural frequencies) and eigenvectors (mode shapes) as follows [39]:

(32) $$\alpha_{ij}\left(\omega \right)=\sum_{r=1}^{N}\frac{\phi_{ir}\phi_{jr}}{\omega_{r}^{2}-\omega^{2}+i\eta_{r}\omega_{r}}$$

Here, *N* is the number of modes, $\phi_{ir}$ and $\phi_{jr}$ are the eigenvectors of the response and excitation coordinates for mode *r*, respectively, $\omega_{r}$ is the frequency of mode *r*, ω is the frequency of the excitation force, and ${ƞ}_{r}$ is the modal damping ratio of mode *r*. In this way, in the most general case, the FRFs in the desired frequency range can be calculated by summing the effects of all modes for each frequency value ω.

**Figure 8:**
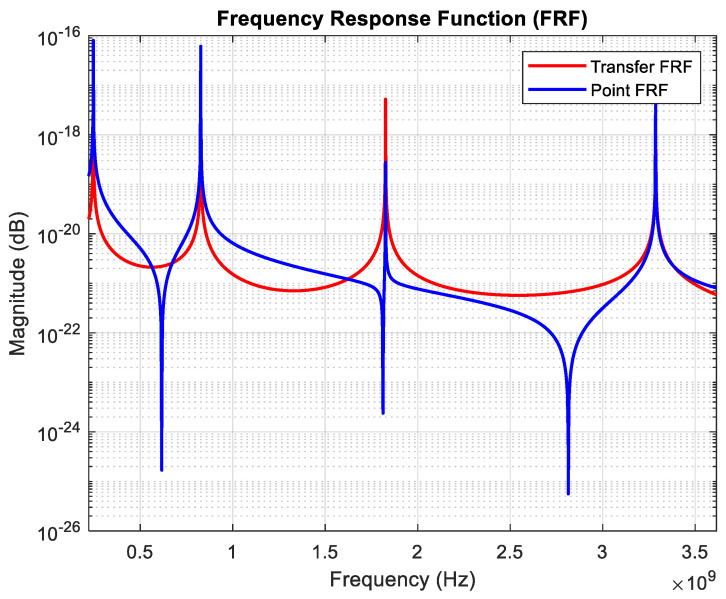
Point and transfer FRFs for *μ*/*L* = 0 and clamped–free boundary conditions.

**Figure 9:**
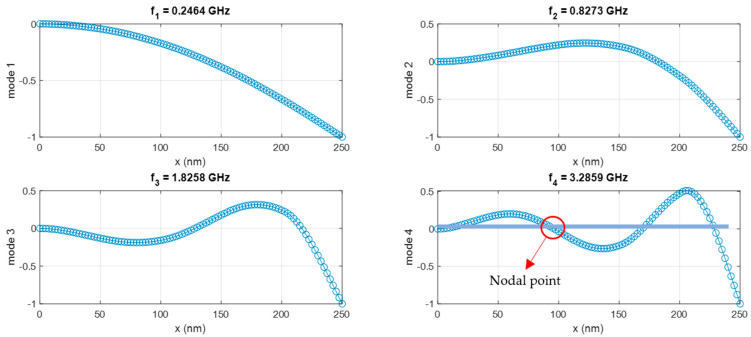
Mode shapes of stepped nanotubes for *μ*/*L* = 0 and clamped–free boundary conditions.

The point and transfer FRFs calculated using Equation (32) for *μ*/*L* = 0 and clamped–free boundary conditions are shown comparatively in Figure 8. The mode shapes of the first four modes are also given in Figure 9.

As seen in Figure 8, an inverse peak occurs between two resonance peaks in the point FRFs. These inverse peaks correspond to the anti-resonance frequencies of the structure. When the structure is excited at its anti-resonance frequencies, the resulting vibration amplitudes remain very low. While resonance frequencies are a general property of the system and, therefore, appear in all FRFs, anti-resonance frequencies are related to the local characteristics of the structure and thus may not occur in every FRF or may vary across them.

In the first mode, the structure exhibits a fundamental bending behavior, where the displacement gradually increases towards the free end, reaching its maximum amplitude at the extremity. In the second mode, a single nodal point can be observed approximately at the mid-span, indicating a higher-order bending pattern. The third mode reveals two nodal points, reflecting a more complex deformation behavior of the structure. Finally, in the fourth mode, three nodal points are observed, where the deformation becomes even more intricate as the natural frequency increases.

The validity and predictive accuracy of nonlocal models are fundamentally dependent on the precise determination of the parameter *μ*. This parameter’s value was obtained by fitting the model’s dispersion curves to the benchmark results generated by atomic models [32,40]. Based on research focusing on the nonlocal analysis of nanostructures like carbon nanotubes, a typical range for the nonlocal parameter, *μ*, is established between 0 and 2 nm [41].

Further extending the analysis of dynamic characteristics, the natural frequencies of the multilayer carbon nanotubes are systematically investigated under various boundary conditions and *μ*/*L* ratios, as detailed in Table 4, Table 5, Table 6 and Table 7. These tables collectively present the calculated natural frequencies for the first few vibration modes across a spectrum of *μ*/*L* ratios (ranging from 0.0 to 2.0) under simply–simply, clamped–free, clamped–clamped, and clamped–simply boundary conditions, respectively. A general trend observed across all tables is a decrease in natural frequencies as the *μ*/*L* ratio increases, indicating that increasing the length of the free end or reducing the fixed length tends to reduce the stiffness and thus the natural frequencies of the nanotube. The specific values provided in these tables are crucial for design considerations, as they highlight the sensitivity of the nanotube’s dynamic behavior to both its geometric configuration (*μ*/*L*) and its support conditions. Such comprehensive data is vital for predicting resonance phenomena, optimizing structural stability, and enabling precise control over the vibrational response of these advanced nanomaterials in diverse engineering applications.

**Figure 10:**
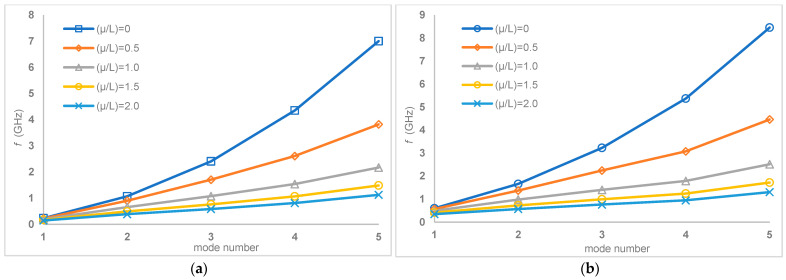
Variation of natural frequencies with respect to *μ*/*L* ratios for (**a**) simply–simply and (**b**) clamped–clamped boundary conditions.

Finally, graphical representations that further explain the variation of natural frequencies with respect to *μ*/*L* ratios at different boundary conditions are presented in Figure 10 andFigure 11.

**Figure 11:**
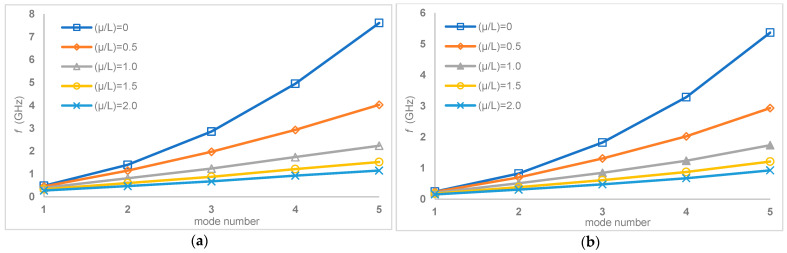
Variation of natural frequencies with respect to *μ*/*L* ratios for (**a**) clamped–simply and (**b**) clamped–free boundary conditions.

Specifically, Figure 10 shows this relationship for simply–simply and clamped–clamped boundary conditions, and Figure 11 for clamped–simply and clamped–free boundary conditions. These figures visually support the trend identified in the tables and show that a consistent decrease in natural frequencies is observed for all tested boundary conditions as the *μ*/*L* ratio increases. This graphical visualization highlights the significant influence of both the length parameter (*μ*/*L*) and the support conditions on the dynamic behavior of multilayer carbon nanotubes, providing a clear and intuitive understanding of how these factors contribute to the overall vibration response and structural stability of the nanostructure.

**Figure 12:**
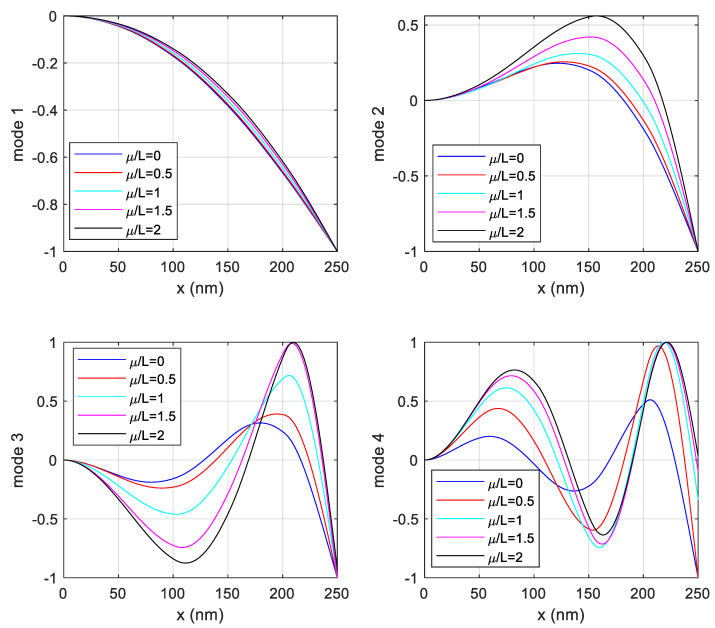
Variation of mode shapes with respect to *μ*/*L* ratios for clamped–free boundary conditions.

Figure 12 shows the variation of mode shapes of the stepped nanotube for the clamped–free boundary conditions for different *μ*/*L* ratios. The first mode corresponds to the fundamental bending mode of the structure; the curves associated with different *μ*/*L* values almost overlap. This indicates that the first mode shape is only marginally influenced by the nonlocal parameter. In other words, for the most basic vibration mode of the structure, there is no significant difference between the classical theory and the nonlocal theory. For the higher modes, as the *μ*/*L* value increases, both the vibration amplitude and the positions of the nodal points change significantly. This demonstrates that the nonlocal effect renders the structure more “flexible” in these modes.

## 4. Conclusions

This study meticulously investigated the dynamic vibrational characteristics of multilayer carbon nanotubes, a critical endeavor for their prospective integration into advanced engineering applications. Employing a robust methodology that combined classical molecular dynamics simulations for accurately determining the bending stiffness of both single-walled and multi-walled atomistic structures with the Euler–Bernoulli beam theory for comprehensive vibration analysis, the research systematically explored the influence of various parameters. This multiscale approach proved effective in bridging the atomic-level behavior with macroscopic structural dynamics, offering a comprehensive understanding of these complex nanomaterials. The use of molecular dynamics simulations, which provide atomic-level details, is crucial for gaining a deeper understanding of the vibrational behavior and tailoring properties for specific applications.

The findings unequivocally demonstrate that the cross-sectional geometry of the carbon nanotube beams significantly influences their vibrational characteristics, affecting both the natural frequencies and mode shapes. The study highlights that variations in diameter and chirality alter the stiffness and mass distribution of the nanotube, leading to changes in the resonant frequencies. Furthermore, the natural frequencies of multilayer carbon nanotubes were systematically investigated under various boundary conditions and *μ*/*L* ratios. A general trend observed in this investigation is a decrease in natural frequencies as the *μ*/*L* ratio increases, indicating that increasing the length of the free end or reducing the fixed length tends to reduce the stiffness and thus the natural frequencies of the nanotube. The specific values obtained for these natural frequencies are crucial for design considerations, as they highlight the sensitivity of the nanotube’s dynamic behavior to both its geometric configuration (*μ*/*L*) and its support conditions. As the nonlocal parameter *μ*/*L* increases, the vibration amplitudes of the nanostructure tend to rise. This implies that the nonlocal theory predicts the structure to be more flexible compared to the classical theory. The magnitude of the nonlocal effect increases significantly with the mode number. While low-frequency (low-order) vibrations are only slightly influenced, high-frequency complex vibrations are strongly affected by nonlocal effects.

Ultimately, the comprehensive data and insights provided by this research furnish invaluable information for the precise prediction of resonance phenomena, the optimization of structural stability, and the effective control over the vibrational response of multilayer carbon nanotubes. Understanding these dynamic behaviors is paramount for their successful engineering implementation, particularly in diverse applications such as mass sensors, actuators, and the enhancement of mechanical properties in composite materials. This work not only contributes to the fundamental understanding of nanoscale mechanics but also provides a crucial foundation for guiding the design and development of innovative nanodevices and materials with tailored dynamic properties, ensuring their reliability and performance in various engineering contexts.

## Figures and Tables

**Table 1:** The geometric parameters of atomistic models.

Model Name	Chirality Vectors	Number of Atoms	Length (nm)	Diameter (Å) [25]	Area (Å^2^) [25]
1 Layer	(5, 10)	6220	50	10.36392	84.360198
2 Layer	(5, 10)/(10, 15)	16,470	50	17.07464	144.61748
3 Layer	(5, 10)/(10, 15)/(15, 20)	30,770	50	23.82734	361.54370
4 Layer	(5, 10)/(10, 15)/(15, 20)/(20, 25)	49,130	50	30.59424	650.77867
5 Layer	(5, 10)/(10, 15)/(15, 20)/(20, 25)/(25, 30)	71,560	50	37.36762	1012.3223

**Table 2:** Mechanical properties of the stepped nanotube.

Model Name	Bending Stiffness (EI)		Density (ρ)
	eVÅ	nN (nm)^2^	kg/m^3^
1 Layer	9131.300	146.288	2938.414
2 Layer	39,956.000	640.113	4538.715
3 Layer	97,930.000	1568.900	3391.773
4 Layer	192,310.000	3080.900	3008.663
5 Layer	326,480.000	5230.400	2817.161

**Table 3:** Natural frequencies for *μ*/*L* = 0 and clamped–free boundary conditions.

Mode Number	Natural Frequencies (GHz)
1	0.246
2	0.827
3	1.826
4	3.286

**Table 4:** Natural frequencies for different *μ*/*L* ratios and simply–simply boundary conditions.

Mode Number	(*μ*/*L*) = 0	(*μ*/*L*) = 0.5	(*μ*/*L*) = 1.0	(*μ*/*L*) = 1.5	(*μ*/*L*) = 2.0
1	2.33 × 10^8^	2.22 × 10^8^	1.97 × 10^8^	1.70 × 10^8^	1.45 × 10^8^
2	1.07 × 10^9^	9.00 × 10^8^	6.56 × 10^8^	4.91 × 10^8^	3.87 × 10^8^
3	2.41 × 10^9^	1.70 × 10^9^	1.08 × 10^9^	7.61 × 10^8^	5.83 × 10^8^
4	4.35 × 10^9^	2.61 × 10^9^	1.54 × 10^9^	1.07 × 10^9^	8.13 × 10^8^
5	7.00 × 10^9^	3.82 × 10^9^	2.17 × 10^9^	1.49 × 10^9^	1.12 × 10^8^

**Table 5:** Natural frequencies for different *μ*/*L* rates and clamped–clamped boundary conditions.

Mode Number	(*μ*/*L*) = 0	(*μ*/*L*) = 0.5	(*μ*/*L*) = 1.0	(*μ*/*L*) = 1.5	(*μ*/*L*) = 2.0
1	6.02 × 10^8^	5.68 × 10^8^	4.93 × 10^8^	4.14 × 10^8^	3.49 × 10^8^
2	1.66 × 10^9^	1.37 × 10^9^	9.80 × 10^8^	7.27 × 10^8^	5.69 × 10^8^
3	3.23 × 10^9^	2.24 × 10^9^	1.40 × 10^9^	9.92 × 10^8^	7.63 × 10^8^
4	5.37 × 10^9^	3.07 × 10^9^	1.79 × 10^9^	1.24 × 10^9^	9.46 × 10^8^
5	8.45 × 10^9^	4.46 × 10^9^	2.52 × 10^9^	1.72 × 10^9^	1.30 × 10^9^

**Table 6:** Natural frequencies for different *μ*/*L* rates and clamped–simply boundary conditions.

Mode Number	(*μ*/*L*) = 0	(*μ*/*L*) = 0.5	(*μ*/*L*) = 1.0	(*μ*/*L*) = 1.5	(*μ*/*L*) = 2.0
1	4.83 × 10^8^	4.56 × 10^8^	3.96 × 10^8^	3.32 × 10^8^	2.79 × 10^8^
2	1.40 × 10^9^	1.15 × 10^9^	8.12 × 10^8^	6.00 × 10^8^	4.70 × 10^8^
3	2.86 × 10^9^	1.97 × 10^9^	1.24 × 10^9^	8.77 × 10^8^	6.76 × 10^8^
4	4.95 × 10^9^	2.93 × 10^9^	1.74 × 10^9^	1.22 × 10^9^	9.29 × 10^8^
5	7.61 × 10^9^	4.03 × 10^9^	2.24 × 10^9^	1.52 × 10^9^	1.15 × 10^9^

**Table 7:** Natural frequencies for different *μ*/*L* rates and clamped–free boundary conditions.

Mode Number	(*μ*/*L*) = 0	(*μ*/*L*) = 0.5	(*μ*/*L*) = 1.0	(*μ*/*L*) = 1.5	(*μ*/*L*) = 2.0
1	2.46 × 10^8^	2.36 × 10^8^	2.10 × 10^8^	1.80 × 10^8^	1.54 × 10^8^
2	8.27 × 10^8^	7.01 × 10^8^	5.13 × 10^8^	3.87 × 10^8^	3.08 × 10^8^
3	1.83 × 10^9^	1.31 × 10^9^	8.48 × 10^8^	6.13 × 10^8^	4.77 × 10^8^
4	3.29 × 10^9^	2.02 × 10^9^	1.24 × 10^9^	8.77 × 10^8^	6.76 × 10^8^
5	5.37 × 10^9^	2.94 × 10^9^	1.74 × 10^9^	1.22 × 10^9^	9.29 × 10^8^

## Data Availability

The data that support the findings of this study are available from the corresponding author upon reasonable request.

## Abbreviations

The following abbreviations are used in this manuscript:

VTGSs	Variable-Thickness Graphene Sheets
MD	Molecular Dynamics
LAMMPS	Large-scale Atomic/Molecular Massively Parallel Simulator
FFT	Fast Fourier Transform
VACF	Velocity Autocorrelation Function
CNTs	Carbon Nanotubes
FEM	Finite Element Modeling
SWNTs	Single-Walled Carbon Nanotubes
SWCNCs	Single-Walled Carbon Nanocones
SLGSs	Single-Layered Graphene Sheets
DLGSs	Double-Layered Graphene Sheets
MWCNTs	Multi-Walled Carbon Nanotubes
vdW	Van Der Waals
VMD	Visual Molecular Dynamics
